# Enhancing hospital protection measures reduces frontline medical workers’ stress during the pandemic

**DOI:** 10.1186/s40359-024-02185-8

**Published:** 2024-12-03

**Authors:** Zhou Xiaoxia, Feng Yan, Wang Junwei, Zhang Bingyao, Xu Fei

**Affiliations:** 1 Longhua Hospital, Shanghai University of Traditional Chinese Medicine, No.725, Wanping South Road, Shanghai, Xuhui Area 200030 China; 2 School of Statistics, East China Normal University, 500 Dongchuan Road, Shanghai, 200241 China; 3 Hubei University of Medicine, No. 30, Renmin South Road, Shiyan City, Hubei Province China

**Keywords:** China, COVID-19, Frontline medical worker, Stress, Factor, Stress trend, Hospital protections

## Abstract

Frontline medical workers (FMWs) faced high stress levels in frontline clinics during the COVID-19 pandemic. This study aims to alleviate FMWs’ psychological stress by enhancing protection measures, which includes refining the selection process, improving protective measures for their work in frontline clinics, and determining the appropriate time for evacuation. A Chinese-language-version stress questionnaire, covering stress-related issues and sociodemographic characteristics, was administered to FMWs in Shanghai during the COVID-19 pandemic in 2022, yielding 107 valid responses. The questionnaire’s reliability and validity were subsequently confirmed. Exploratory factor analysis (EFA) was applied to the stress-related questions, with the KMO and Bartlett’s test values of 0.886, 877.8522 (df = 120, *p* < 0.0001), respectively. Structure equation modeling (SEM) was used to confirm the factors and measure the relationships among the stress-related questions, factors and sociodemographic characteristics. The parameters of SEM named SOFAIDC were significant with SRMSR, RMSEA, AGFI, NFI, and PNFI being 0.0422, 0.0507, 0.9946, 0.9958, and 0.7615, respectively. Paired sample t-tests revealed the significant decrease in FMWs’ stress score of the current week and the previous week, with all t-values being negative and significant (*p* < 0.01). A latent growth model (LGM) named LGM30 verified the overall decreasing stress trend over the weeks, with high R-squares (> 0.80), significant positive linear and negative quadratic time parameters. Based on the trajectory analysis, a stress scorecard with an AUC of 0.856 was developed. This study found that there are four factors for FMWs’ psychological stress (anxiety about infection, exhaustion, lack of cognition for infection, and hesitant feeling), and improving targeted hospital protection could alleviate FMWs’ psychological stress through SOFAIDC, as all the total effect of factors or items from the feeling of being protected by hospital were negative. Meanwhile, although overall FMWs’ stress trend has decreased, 22.43% of FMWs have maintained or experienced a high stress trend, and the stress might increase over weeks in frontline clinics. To perfect hospital protection to alleviate FMWs’ psychological stress, the scorecard could be used to select FMWs with low-stress trends, LGM30 could be used to determine the withdrawal time (such as week 8), and targeted hospital protections could be designed based on the relationships between feeling of being protected by the hospital and factors in SOFAIDC, such as providing adequate protective equipment, improving the rationality of FMWs’ work schedules, recruiting personnel with higher education levels, and paying adequate monetary compensation.

**Supplementary Information:**

The online version contains supplementary material available at 10.1186/s40359-024-02185-8.

## Introduction

Since the beginning of the 21st century, humans have faced multiple epidemics, including SARS, H1N1, and COVID-19, and are likely to encounter more in the future. These waves of viral outbreaks continuously pose unprecedented challenges for health systems and medical workers (MWs).

The phenomenon of increased work stress and job insecurity has been even more obvious among frontline medical workers (FMWs), as they have been in the front line of prevention and control since the outbreak of COVID-19 [29, 70]. Within the frontline clinics, FMWs are at a higher risk of infection for themselves and their co-residents; they are also constantly fearful of being infected [5, 11, 47, 51, 56, 84]. Literature shows that cognitive appraisals can impact perceived self-efficacy and psychological distress during high-stress situations such as COVID-19 lockdowns [17, 19].

The severe situation is causing mental health problems such as stress, anxiety, depressive symptoms, insomnia, denial, anger, and fear [25, 27, 40, 75, 77]. The physical and mental effort involved in caring for patients with COVID-19 has caused acute stress, compassion fatigue, and other affective pathologies, which, together with psychosomatic reactions, have affected work morale [31, 44, 45, 49]. Challenge stress and hindrance stress have a significant impact on presenteeism (i.e. lost productivity or reduced performance) among nurses [37]. Stress and burnout can have several negative effects on the worker’s work performance, along with their mental health and well-being [1]. Therefore, the severe situation in frontline clinics directly causes the psychological stress among FMWs, and this psychological stress can reduce FMWs’ work performance and mental health.

During the COVID-19 pandemic, 63% of 632 participants in Canada and the United States reported a significantly high level of stress [85]. Health center workers (HCWs) experienced significantly higher work stress than the general population [72, 81]. The lowest (29.8%) and highest (62.99%) reported prevalence of stress among MWs were noted, and MWs in areas with higher infection rates reported more severe degrees of all psychological symptoms than other health care workers [86, 88], indicating that FMWs were more prone to psychological stress.

For FMWs, stress causes worries, anxiety, and depression, which affects their mental and physical health as well as their patient-care performance [54]. Prolonged high stress levels can produce adverse health outcomes when unaddressed [53]. FMWs face physical and psychological stresses caring for critically ill patients, including experiencing anxiety, depression, and posttraumatic stress symptoms [3]. 76.5% of the 376 participants who have been exposed to stressful situations and increased workload during the COVID-19 pandemic had scores compatible with a diagnosis of traumatic stress disorders [87]. Stress is higher among nursing assistants, medical assistants, inpatient workers, which is related to workload and mental health, and is lower when feeling valued [69]. The high prevalence of depression, anxiety and stress is attributed to the various sources of workplace worries and the inappropriate coping strategies among FMWs, and measures that minimize workplace worries and inappropriate coping strategies must be implemented promptly [38]. It is necessary to diagnose factors that increase the stressfulness of health workers’ work, so that effective actions to counteract them can be taken [70]. Therefore, interventions should be implemented to reduce FMWs’ psychological stress.

Taking no action to address the burden of major depressive disorder and anxiety disorders should not be an option, and mitigation strategies could incorporate ways to promote mental wellbeing [12]. In the general workspace, the employer-sponsored mental health program is associated with large clinical improvements in depression and anxiety, and a positive financial return on investment (ROI) across all employee salary levels; However, it has not established causality between the improvements and the care provided [3]. Targeted, well-designed mental health interventions can improve outcomes among health care workers [90]. Thus, targeted interventions for reducing FMWs’ psychological stress should be designed and implemented.

To our knowledge, although hundreds of studies have examined mental health of FMWs during the COVID-19 pandemic and other pandemics [2, 6, 13, 18, 26, 27, 31, 60, 70, 92, 93], these studies focused on different aspects of psychology of FMWs or MWs, such as anxiety, stress, sleep, or revealed the associations between psychological indices and socioeconomic characteristics. However, there has been no published research exploring systematic protective measures to reduce the psychological stress of FMWs; for example, which MWs should be prioritized for frontline clinics, the development and implementation of mental health protection based on the impact path of FMWs’ psychological stress in frontline clinics, and when FMWs should be withdrawn to ensure their mental health.

The aim of this study is to alleviate FMWs’ psychological stress related to COVID-19 pandemic by enhancing protection measures, which includes refining the selection process, improving protective measures for their work in frontline clinics, and determining appropriate time for evacuation. Firstly, an appropriate and previously published psychological scale should be selected and translated into Chinese. The questionnaire should be distributed and the data should be collected. Secondly, the reliability and validity of the psychological scale should be measured and verified. Thirdly, the factors of FMWs’ psychological stress should be explored and confirmed, and external and implementable variables that can influence these factors should be identified to improve protections and alleviate FMWs’ psychological stress while working in frontline clinics. Lastly, the overall stress trend should be analyzed to determine when the FMWs should be evacuated from frontline clinics, and the stress trend of each FMW should be distinguished and verified to determine who has priority to work in the frontline clinics.

## Methods

A two-stage methodological and validation study was conducted, with Stage 1 focusing on the selection and translation of a questionnaire to measure the stress of FMWs, and Stage 2 focusing on the psychometric properties of the translation.

### Stage 1. Selection and translation of questionnaire process

#### Step 1. Selection of questionnaire

The study focused on a sample population of nurses and doctors serving in Shanghai’s frontline clinics during the COVID-19 pandemic from March to June 2022. Amidst the city-wide lockdown during the period, there was a significant shortage of daily necessities and essential medicines. As a result, the stress experienced by FMWs was multifaceted encompassing not only the anxiety, insomnia, burnout, and exhaustion associated with their frontline clinics duties but also the strain of fulfilling family responsibilities amidst these challenging time.

According to the previously published studies, many studies confirmed that perceived stress during the pandemic was positively relevant to individuals’ emotional response such as anxiety and depression [71, 96], physical responses such as insomnia [98], and the increase in work stress was associated with job-related burnout [72]. For FMWs, some common sources of stress included exposure to infectious diseases, heavy workloads, facing ethical dilemma in clinical decision-making, and unfamiliar problems from the pandemic [55]. The content of the questionnaire should include anxiety, depression, insomnia, and burnout.

There were many psychological scales for assessing the psychological stress of FMWs, such as IES [54, 66, 67, 94], DASS-21 [83], and HEDQ [48, 52] among others. The stress-related questions associated with the H1N1 event [59] were found to significantly correspond with the findings of previous studies about FMWs’ stress for working at frontline clinics in COVID-19 pandemic. The stress-related questions associated with the H1N1 event were based on the items in studies on severe acute respiratory syndrome (SARS), hypothetical influenza and H1N1 pandemic [59]. Hence, the stress-related questionnaire was chosen as a measurement tool for FMWs’ stress.

#### Step 2. Translation of questionnaire

One Chinese-speaking translator with proficiency in English translated the original English psychological scale into Chinese. The translator was instructed to create an acceptable and natural version of the scale, ensuring simplicity, clarity, and relevance for the general Chinese-speaking population.

#### Step 3. Backward translation and adjustment

At this step, the Chinese version of the stress-related questionnaire was back-translated into English by additional translators. In case of discrepancies between the back-translation and the original scale, a reiteration was initiated with re-reviewing the forward translation, ensuring corrections to the translation. If any differences did not warrant another cycle of translation and review, the process proceeded to the next stage.

#### Step 4. The contents of questionnaire

The English version of the stress-related questionnaire contains 20 psychological stress characteristics as items *T1*-*T20* (see Table 1). *T1*-*T11* and *T13*-*T17* were based on the items in studies on severe acute respiratory syndrome (SARS) and hypothetical influenza pandemics, while *T12*, *T18*, and *T19*were based on incentives to work during the H1N1 pandemic [59]. We have added an original item about the inability to fulfill family responsibilities while working at the frontline clinics during COVID-19 pandemic (*T20*).

**Table 1:** Descriptive statistics and Cronbach's alpha coefficient for variables

Variable	Mean(±Std) / frequency (%)	Correlation with Total	Cronbach's Alpha coefficient
T1 **Anxiety about being infected**	2.78±0.79	0.5856	0.8007
T2 **Anxiety about infecting family**	2.75±0.89	0.4318	0.8082
T3 **Burden of change of quality of work**	2.75±0.89	0.6368	0.7965
T4 **Burden of increase quantity of work**	2.84±0.88	0.5995	0.7988
T5 **Anxiety of being infected during commuting**	2.58±0.81	0.6627	0.7964
T6 **Lack of knowledge about prevention and protection from infection**	1.88±0.72	0.6272	0.7999
T7 **Lack of knowledge about infectivity and virulence**	1.97±0.72	0.6415	0.7993
T8 **Feeling of being avoided by others**	1.8±0.85	0.5331	0.8028
T9 **Feeling of being protected by national and local governments**	3.29±0.82	-0.2674	*0.8420*
T10 **Feeling of being protected by hospital**	3.54±0.66	-0.3912	*0.8410*
T11 **Anxiety about compensation**	2.36±1	0.5572	0.8003
T12 **Hesitation to work**	1.7±0.72	0.5771	0.8023
T13 **Feeling of being isolated**	1.74±0.85	0.5543	0.8017
T14 **Elevated mood**	2.72±0.81	-0.2399	0*.8402*
T15 **Sleep disturbances**	2.57±0.93	0.3511	0.8129
T16 **Mental exhaustion**	3.06±0.75	0.5737	0.8019
T17 **Physical exhaustion**	2.87±0.84	0.5960	0.7996
T18 **Motivation to work**	3.41±0.66	-0.2719	*0.8365*
T19 **Feeling of having no choice but to work due to obligation**	2.02±1.03	0.3970	0.8106
T20 **Burden of being unable to take care of family members due to work**	2.91±0.97	0.5836	0.7988
T22 time length at frontline clinics	2.33±14.14	-	-
**Gender**^a^		-	-
male	14(13.08%)		
female	93(86.92%)		
**Age(year)**^a^		-	-
21-25	13(12.15%)		
26-30	33(30.84%)		
31-35	29(27.1%)		
36-40	22(20.56%)		
41-45	6(5.61%)		
46-50	2(1.87%)		
51-60	1(0.93%)		
60 or above	1(0.93%)		
**Education level**^a^		-	-
High school/technical secondary school	1(0.93%)		
Junior college	20(18.69%)		
undergraduate	70(65.42%)		
postgraduate	16(14.95%)		
**Working year(year)**^a^		-	-
0-5	23(21.5%)		
6-10	35(32.71%)		
11-15	27(25.23%)		
16-20	13(12.15%)		
20 above	9(8.41%)		
**Profession**^a^		-	-
physician	17(15.89%)		
Nurse	90(84.11%)		

^a^frequency(percent) was displayed

The respondents used a 4-point scale (1, never; 2, rarely; 3, sometimes; 4, always) to describe how they felt and experienced the 20 items during their time at the frontlines fighting the COVID-19 epidemic.

Furthermore, FMWs’ psychological responses were significantly associated with sociodemographic characteristics, such as age, gender, profession, education level, experience (or working years), checking the authenticity of information and the frequency of such check, where the frequency of checking the authenticity of information could be replaced by the length of time to complete the questionnaire. Hence, age, gender, education level, working years, and profession should be basic components of the questionnaire.

### Stage 2. Psychometric evaluation of the Chinese version of questionnaire

#### Step 1. Sample and sampling method

Random sampling was performed. The sample size was determined based on the sample size of factor analysis and structural equation modeling (SEM). The sample size of factor analysis was in the range of 100 to 200 when the communalities are in the range of 0.5 [39]. Meanwhile, the sample size of SEM was in the range of 30 to 240, and the required sample size would decrease when the number of indicators of a factor increases [89]. Thus, in this study, in order to increase the stability and accuracy of the results, 100 participants should be randomly sampled.

However, considering that some of the collected questionnaires may be invalid, the stress-related questionnaires in Chinese were randomly distributed online to 150 FMWs who were fighting the COVID-19 pandemic at the frontline clinics in Shanghai in 2022, resulting in 107 valid responses (71.3%) who had continuously self-rating their own stress and provided feedback. The sociodemograpical characteristics of the FMWs and their responses to the stress-related questions in the Chinese version of questionnaire were listed in Table 1.

#### Step 2. Reliability analysis

The Cronbach’s alpha coefficient of 16 items based on SARS and hypothetical influenza pandemic was 0.7914, indicating the items need to be reviewed. The Cronbach’s alpha coefficient of the 19 items without item *T20* was 0.7988, indicating that these items also need to be reviewed. The Cronbach’s alpha coefficient of the 20 items for the Chinese version of the stress-related questions was 0.8184, indicating good consistency and acceptable reliability.

The Cronbach’s alpha coefficients with deleted variables are in Table 1. The coefficients of *T9*, *T10*, *T14* and *T18* were 0.8420, 0.8410, 0.8402, 0.8365, respectively, all of which were greater than 0.8184.Therefore, these four items should be deleted. The Cronbach’s alpha coefficient of the 19 items without items *T9*, *T10*, *T14*, *T18* and *T20* was 0.8960, indicating good consistency and acceptable reliability. The Cronbach’s alpha coefficient of the 20 items without items *T9*, *T10*, *T14* and *T18* was 0.9028, which entails that the Chinese version of the stress-related questions had good consistency and acceptable reliability.

#### Step 3. Validity analysis

The content validity of the Chinese version of the stress-related questions has be verified by some studies. The majority of MWs were concerned with the risk of their health [11, 32, 57, 91–93], which indicates*T1*,* T5*, *T12*, and *T18*should be included. The most frequent concern was the infection of family and friends and the health consequences of the disease [15, 57, 91–93], which shows*T2*should be included. The majority of MWs expected an increased workload and to feel more stressed at work [7, 57, 58, 62, 91, 93], which means*T3* and *T*4 should be included. The risk perception and knowledge gaps were as barriers of pandemic influenza response [6, 16, 18], which means*T6* and *T7*should be included. The avian pandemic adverse impacts on MWs’ personal life and work, such as people avoiding them [59, 82], which indicates*T8* and *T13* should be included for FMWs’ stress.

The childcare responsibilities, prioritizing the wellbeing of family members, lack of trust in the health system, and lack of information about the risks were barriers that prevent MWs from performing their obligations [20, 32, 35, 36], which shows*T9*, *T10*, *T19*, and *T20*should be factors measuring stress. In the COVID-19 pandemic, FMWs experienced a range of mental health symptoms primarily related to perceptions of institutional betrayal (inadequate staffing and resourcing) as well as feeling unable to fulfill their duty of care towards patients [30, 31], which indicates FMWs feeling burnout means that*T11*, *T16*, and *T17*should be stress indicators. FMWs reported significantly higher scores on Pittsburgh Sleep Quality Index (PSQI) and anxiety, and a higher prevalence of sleep disturbances, [27, 70], which means that*T14* should be a stress indicator.

The criterion validity of the 16 items was confirmed. The correlation between the total score of the 16 items and the IES score was 0.35 [59], indicating the validity is acceptable. The structure validity of the questionnaire was demonstrated because the KMO test was greater than 0.8 and*p*-value for Bartlett’s test was less than 0.0001.

Additionally, the structure validity of the stress-related questions in Chinese was evaluated by explore factor analysis (EFA) with 20 items (in Supplemental Table 1), which shows that item *T9*, *T10*, *T14* and *T18* should be deleted to ensure the validity of the questionnaire. The structure validity of the 20 items without item *T9*, *T10*, *T14* and *T18* was evaluated by explore factor analysis.

Therefore, the items of the Chinese version of the stress-related questions should be *T1*-*T8*, *T11*-*T13*, *T15*-*T17*, and *T19*-*T20* for psychometric properties to ensure its reliability, validity, and consistency.

### Statistical analysis

Based on reliable, valid and consistent questionnaire data, some statistical analyses were carried out using SAS 9.2.

### Descriptive analysis

86.92% of the participants are female, and 84.11% are nurse. 78.5% of the participants are aged between 26 and 40. 80.3% of the participants have a bachelor’s or master’s degree. 78.5% of the participants have worked more than 5 years, and the average time length during the frontline clinics for the participants was 2.33 weeks. Table 1 shows the descriptive values of the variables used in this study.

For the Chinese version of the stress-related questions, the average items *T1*, *T2*, *T5*, and *T11* were 2.78,2.75, 2.58, and 2.36, indicating that FMWs sometimes feel anxious. The average items *T3*, T*4*, and *T20* were 2.75, 2.84, and 2.91, indicating that the participants sometimes feel burdened. The average items *T6*, *T7*, *T8*, *T12*, and *T13* were 1.88, 1.97,1.8, 1.7, and 1.74, indicating that the participants rarely feel a lack of knowledge and self-efficacy. The average items *T15*, *T16*, *T17*, and *T19* were 2.57, 3.06, 2.87, and 2.02, indicating that the participants seldom feel exhaustion.

As shown in Supplemental Fig. 1, the Kendall correlations among the items and sociodemographic characteristics are displayed. We observed that the sociodemographic characteristics were highly correlated, but their relevance to items of the Chinese version of the stress-related questions was relatively low.

### Exploratory factor analysis for reveal the factors

The structure and factors of the Chinese version of the stress-related questions for FMWs’ stress were revealed via Exploratory factor analysis (EFA) with the maximum likelihood method and bivarimax rotation. Table 2 shows the result of EFA in this study.

**Table 2:** Factor analysis of the 16 stress-related questions

**Factors**	**Questions**	***f1***	***f2***	***f3***	***f4***	**KMO**	**Communality**
***Factor1*:** **Anxiety of infection** **(Cronbach's α=0.8181)**	*T1*	***0.80***	0.26	0.20	0.11	0.83	0.77
	*T2*	***0.68***	0.10	0.15	0.19	0.87	0.53
	*T5*	***0.62***	0.31	0.31	0.20	0.91	0.62
***Factor2*:** **exhaustion** **(cronbach's α=0.8250)**	*T3*	0.34	***0.56***	0.25	0.08	0.89	0.49
	*T4*	0.25	***0.65***	0.33	-0.07	0.89	0.59
	*T15*	-0.03	***0.42***	0.06	0.32	0.84	0.29
	*T16*	0.07	***0.84***	0.09	0.17	0.83	0.75
	*T17*	0.16	***0.79***	0.10	0.21	0.84	0.71
	*T20*	0.30	***0.41***	0.19	0.27	0.94	0.37
***Factor3*:** **Lack of cognition for infection** **(Cronbach's α=0.7582)**	*T6*	0.17	0.13	***0.96***	0.19	0.78	1.00
	*T7*	0.20	0.22	***0.80***	0.18	0.79	0.76
	*T19*	0.15	0.20	***0.40***	0.11	0.88	0.24
***Factor4*:** **hesitant feeling** **(Cronbach's α=0.767)**	*T8*	0.24	0.26	0.35	***0.37***	0.92	0.39
	*T11*	0.21	0.23	0.31	***0.66***	0.92	0.63
	*T12*	0.30	0.25	0.40	***0.44***	0.94	0.51
	*T13*	0.27	0.29	0.17	***0.59***	0.91	0.54
**Squared multiple correlations of the variables with each factor**		0.85	0.99	0.79	0.66		
**Unweighted variance explained by each factor**		2.96	2.52	2.16	1.55		
**Variance explained**		32.25%	27.40%	23.48%	16.87%		
**H0: 3 factors are sufficient H1: More factors are needed**		114.2528(df=75, *p*=0.0024)					
**H0: 4 factors are sufficient H1: More factors are needed**		72.0602(df=62, *p*=0.1793)					
**Tucker & Lewis Reliability coefficient**		0.9743					
**Kaiser-Meyer-Oklin(KMO) test for model**		0.8699					
**Bartlett's test of sphericity**		877.8522(df=120, *p*<0.0001)					
**Correlation between factors**							
	F2	-0.004					
	F3	0.061	0.071				
	F4	0.052	-0.031	-0.053			

The value of *Kaiser-Meyer-Olkin (KMO)* test for the complete model is 0.8699, which is greater than 0.8, indicating that the sampling is adequate for EFA. The values of *KMO* test for each variable in the model are greater than 0.78 which means the sampling adequacy for each variable is meritorious. Hence, the EFA could be applied.

The chi-square of significance test for 3 factors being sufficient was 114.2528 (df = 75, *p* = 0.0024), indicating that more than 3 factors are needed. The chi-square of significance test for 4 factors being sufficient was 72.0602 (*p* = 0.1792), indicating 4 factors are sufficient. The variance explained also indicated 4 factors should be used.

The measure of congruence coefficient (K) for EFA [39] with 4 factors is 0.9049, which is greater than 0.9, indicating a good correspondence between sample and population factors.

Although the correlations among factors were less than 0.1 and not significant, there are four factors which means second-order factor analysis should be applied for the data. Additionally, the factor loadings of *T3*, *T4*, *T5*, *T8*, *T11*, *T12*, *T15*, *T16*, and *T20* were greater than 0.3 in two or more factors, which indicates second-order confirmatory factor analysis should be warrants.

### Structural equation modeling for expressing the relationships

Multiple regressions were conducted to analyze the relationship between sociodemographic characteristics and factors derived from EFA. However, the regression coefficients were not statistically significantly, and the R-squared values were less than 0.1.

As an extension of factor analysis and multiple regression, SEM that traditionally hypothesize a set of linear relationships between the observed indicator variables and the latent factors, involves confirmatory factor analysis. SEM was applied to confirm the factors of the Chinese version of the stress-related questions, and to measure the relationships among the factors, indicators for factor and sociodemographic characteristics, with the aim of identifying implementable variable(s) to reduce FMWs’ stress.

The data type of the Chinese version of the stress-related questionnaire for FMWs’ stress was categorical, and polychoric correlation matrix was utilized for SEM with unweighted least squares (ULS). Although ULS and diagonally weighted least squares (DWLS) can produce unbiased parameter estimates and perform best in small and medium samples, ULS tends to perform slightly better [23, 49, 78].

The assessment of fit for SEMs (as shown in Supplemental Table 2) indicated that the factors of psychological stress derived from factor analysis did not fit the data well, based on the recommendation [41]. This was because the GFI, AGF [8, 47], CFI [4, 9, 63, 97], NFI [21, 22, 97] and NNFI were all below 0.9, the SRMSR and RMESEA [63, 97] were above 0.1, the PNFI [10] and PGFI [64] were below 0.5.

Initially, a second-order factor was added to the four factors of psychological stress in SEM, but the assessment of fit (in Supplemental Table 2) indicated that RMSR and SRMSR were greater than 0.05. On this basis, the exogenous variable (*T10*) was added to the four factors and the common factor in the SEM, but the assessment of fit (in Supplemental Table 2) showed that RMSR and SRMSR were greater than 0.05. Based on these findings, the structure was modified to include only those factor loadings greater than 0.3 in SEM. The chi-square, GFI, AGFI, SRMSR, RSMR, RMSEA, PCF, CFI, NFI, NNFI, PNFI, and PGIF of the second-order factor analysis and association among factors, items and sociodemographic characteristics (SOFAIDC) were 31.93(df = 117, *p*-value = 1), 0.9963, 0.9946, 0.0422, 0.0422, 0.0507, 1, 1, 0.9958, 1.0148, 0.7615, and 0.7619, respectively. These results indicate that the SEM named SOFAIDC could be acceptable and suitable for expressing the relationships among the variables.

### Paired T-test and latent growth for stress trends

The questionnaire collected the FMWs’ weekly self-rating scores in the frontline clinics. To verify the hypothesis that the stress levels of the FMWs decreased from the first week to the sixth week, paired sample t-tests were conducted to compare the difference between FMWs’ self-rating scores in current week and previous week, and all t-values were negative and significant (*p* < 0.01), indicating a decreasing trend in the FMWs’ stress trend levels.

Latent growth curve modeling (LGM) was used to evaluate the latent growth of stress trends. Four stress trends were recognized, and the parameters of the models were estimated. To test whether the random effects, stress self-rating scores’ linearly or quadratically effect over time were significant, several LGMs were developed, and the assessments of 31 key LGMs were displayed (see Supplemental Table 3). The results showed that the Schwartz Bayesian Criterion (SBC) of LGM19 was less than that of LGM4 which was less than that of LGM2, indicating the linearly and quadratic effects of stress self-rating scores over time were significant.

Based on the indices of assessment for LGMs, LGM28 could fit the data well, but not all of the parameters were significant (see Supplemental Table 4). LGM30 was parsimonious, with R-square values greater than 0.80, and all parameters significant (see Supplemental Table 5), indicating that LGM30 could fit the data well.

### Trajectory analysis and scorecard for distinguishing stress trends

The time-series of weekly stress self-rating scores was analyzed via trajectory [42] to identify ‘high’ or ‘low’ stress trends for the stress scorecard. The BIC, AIC, Bayesian factor of trajectories analysis with different groups were displayed (see Supplemental Table 6), which showed that the 4-class model could fit the data well.

Fortunately, MWs’ stress status and stress trends were not used as criteria for determining assignments to frontline clinics. Instead, these factors served as the basis for collecting data to develop a stress scorecard, which could assess whether MWs had a low stress trend while working in frontline clinics.

Sociodemographic characteristics and stress-related items could be used for the stress scorecard. The step-by-step process for building a stress scorecard could refer to that of a credit scorecard. According to the step-by-step process of credit scorecards [73], these variables should be binned because the binning process can maintain the stability of variables and transform the 4-point scale values into real numbers that can be used in Logistic regression (LR) to build the stress scorecard. Based on binned data and cases with ‘high’ or ‘low’ stress tends, the Weight of Evidence (WOE) and Information Value (IV) for each bin of a variable would be calculated, and the sum of the IV of each bin of the variable could be calculated to determine whether the variable should be a candidate for LR.

Generally, variables should be used in the stress scorecard when the IV of variables is greater than 0.02. However, more correlated variables would be included in the fit model (see Supplemental Fig. 1). Highly correlated variables would be selected based on the knowledge of stress-related factors Stepwise selection was applied to determine the variables and picked up 7 variables—*Work_year*, *Education level*, *T1*,*T8*, *T14*, *T15* and *T20—* for inclusion in the LR for the stress scorecard.

The area under receiver operating characteristic curve(AUC) was 0.856 and the statistics of Kolmogorov-Smirnov test(KS) was 0.68 (in Supplemental Fig. 3). The greater the area between the Lift / Gain and Baseline, the better the model. By contacting only 10% of medical workers based on the predictive model, it will reach 3.5 times as many respondents, as if it uses no model (in Supplemental Fig. 4). Hence, the anxious model could be used for hospital management to arrange MWs.

## Results

### Four main factors of FMW’s stress

The results of EFA are in Table 2. *Factor1* contains three items, which were about anxiety of being infected (factor loadings of *T1*,* T2*, and *T5* are greater than 0.6) and concluded as ‘anxiety about infection’. The squared multiple correlation of the three items with *Factor1* was 0.85, indicating the meritorious relationship between the three items (*T1*,* T2*, and *T5*) and *Factor1*. The variance explained was 32.25%, which means *Factor1* was a very important factor.

*Factor2* contains six items which were about exhaustion (factor loadings of *T3*, *T4*,* T15*,* T16*,* T17*, and *T20* are greater than 0.4) and concluded as ‘exhaustion’. The squared multiple correlation of the three items with *Factor2* was 0.99, indicating the marvelous relationship between the six items (*T3*, *T4*,* T15*,* T16*,* T17*, and *T20*) and *Factor2*. The variance explained was 27.4%, which means *Factor2* was an important factor.

*Factor3* included three items which were about lack of knowledge for infection (factor loadings of *T6* and *T7* are greater than 0.8) and concluded as ‘lack of cognition for infection’. The squared multiple correlation of the three items with *Factor3* was 0.79, indicating the middling relationship between the three items (*T6*, *T7*, and *T19*) and *Factor3*. The variance explained was 23.48%, which means *Factor3* was an important factor.

*Factor4* included four items which were about hesitance (factor loadings of *T11* and *T13* are greater than 0.35) and concluded as ‘hesitant feeling’. The squared multiple correlations of the three items with *Factor4* was 0.66, indicating the mediocre relationship between the four items (*T8*, *T11*, *T12*, and *T13*) and *Factor4*. The variance explained was 16.87%, which means *Factor4* was a general factor.

### The relationships among items and factors of FMWs’ stress

**Fig. 1:**
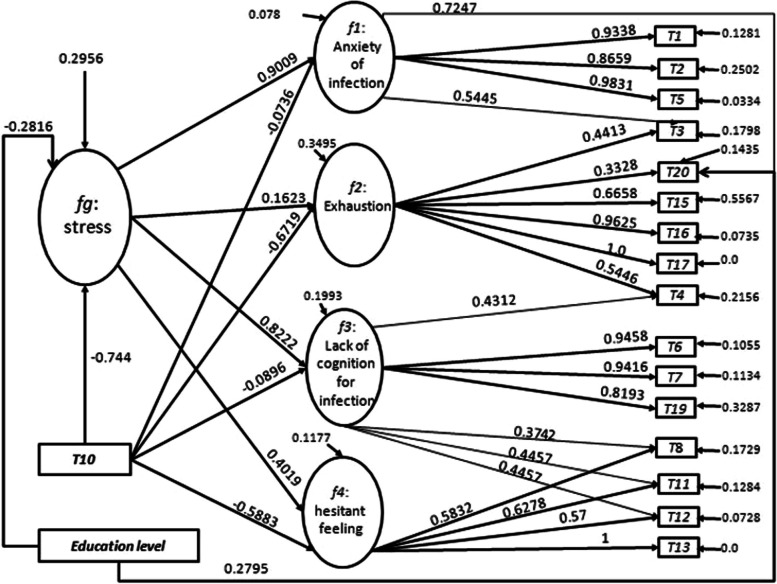
displays the relationships in SEM named SOFAIDC. Ellipses represent latent variables, and rectangles represent observable variables. The single straight arrow indicates a linear relationship from the base to the head of the arrow. Error terms for a variable are represented in the path diagram with an arrow from the error term to the associated variable. All parameter estimations are standardized coefficients

In Fig. 1, SOFAIDC had four latent variables *f1*-*f4* that are correspond to factors *Factor1 to Factor4* from factor analysis, and one second-factor *fg*that is identified as psychological stress, because psychological stress is likely to be a universal risk factor for anxiety, depression, and burnout [1, 33, 55], and the parameters between*f1*-*f4* and *fg* showed that the second-order factor is likely to be stress because parameters between *f1*-*f4* and *fg* were positive (*fg* in *f1*,* f2*,* f3*, and *f4*: $\:\beta\:$=0.9009, 0.1623, 0.8222, and 0.4019), and the total effect of *f1*-*f4* from *fg* were all positive (see Table 3), indicating that *fg* could significantly increase anxiety, exhaustion, lack of cognition for infection, and hesitant feeling.

**Table 3:** Total Effect (direct effect) of variables in SEM named SOFAIDC

	f1	f2	f3	f4	fg	education level	T10
*T1*	0.934(0.934)	0(0)	0(0)	0(0)	0.841(0)	−0.237(0)	−0.695(0)
*T2*	0.866(0.866)	0(0)	0(0)	0(0)	0.78(0)	−0.22(0)	−0.644(0)
*T3*	0.545(0.545)	0.441(0.441)	0(0)	0(0)	0.562(0)	−0.158(0)	−0.755(0)
*T4*	0(0)	0.545(0.545)	0.431(0.431)	0(0)	0.443(0)	−0.125(0)	−0.734(0)
*T5*	0.983(0.983)	0(0)	0(0)	0(0)	0.886(0)	−0.25(0)	−0.731(0)
*T6*	0(0)	0(0)	0.946(0.946)	0(0)	0.778(0)	−0.219(0)	−0.663(0)
*T7*	0(0)	0(0)	0.942(0.942)	0(0)	0.774(0)	−0.218(0)	−0.66(0)
*T8*	0(0)	0(0)	0.374(0.374)	0.583(0.583)	0.542(0)	−0.153(0)	−0.78(0)
*T11*	0(0)	0(0)	0.347(0.347)	0.628(0.628)	0.538(0)	−0.152(0)	−0.801(0)
*T12*	0(0)	0(0)	0.446(0.446)	0.57(0.57)	0.596(0)	−0.168(0)	−0.818(0)
*T13*	0(0)	0(0)	0(0)	1(1)	0.402(0)	−0.113(0)	−0.887(0)
*T15*	0(0)	0.666(0.666)	0(0)	0(0)	0.108(0)	−0.03(0)	−0.528(0)
*T16*	0(0)	0.963(0.963)	0(0)	0(0)	0.156(0)	−0.044(0)	−0.763(0)
*T17*	0(0)	1(1)	0(0)	0(0)	0.162(0)	−0.046(0)	−0.793(0)
*T19*	0(0)	0(0)	0.819(0.819)	0(0)	0.674(0)	−0.19(0)	−0.575(0)
*T20*	0.725(0.725)	0.333(0.333)	0(0)	0(0)	0.707(0)	0.08(0.28)	−0.803(0)
*f1*	0(0)	0(0)	0(0)	0(0)	0.901(0.901)	−0.254(0)	−0.744(−0.073)
*f2*	0(0)	0(0)	0(0)	0(0)	0.162(0.162)	−0.046(0)	−0.793(−0.672)
*f3*	0(0)	0(0)	0(0)	0(0)	0.822(0.822)	−0.232(0)	−0.701(−0.09)
*f4*	0(0)	0(0)	0(0)	0(0)	0.402(0.402)	−0.113(0)	−0.887(−0.588)
*fg*	0(0)	0(0)	0(0)	0(0)	0(0)	−0.282(−0.282)	−0.744(−0.744)

Additionally, SOFAIDC showed that *T10* (Feeling of being protected by hospital) could relieve FMWs’ stress because the parameters were negative (*T10* in *f1*,* f2*,* f3*,* f4*, and *fg* linear equations: $\:\beta\:$=−0.0736, −0.6719, −0.0896, −0.5883, and − 0.2816), and the total effects of stress-related questions and *f1*-*f4* from *T10* were all negative (see Table 3). *T10* was the exogenous causal variable which could change both the direction (positive or negative) and strength of the stress-related questions, *f1*-*f4*, and *fg*. Moreover, *T10* and *T9* had a high positive correlation coefficient. Furthermore, *T9* was the indirect exogenous causal variable because the protections from the state may be channeled through hospitals to benefit FMWs.

Education level was another variable that could reduce second-order factor because the parameter was negative (*education level* in the *fg* linear equation: $\:\beta\:$=−0.2816 in SOFAIDC), indicating that the higher the education level, the lower the psychological stress. The education level could also change both the direction (positive or negative) and strength of the stress-related questions, *f1*-*f4*, and *fg*, because their total effects from education level were all negative except the *T20* whose total effect from education level was positive (see Table 3). However, gender, age, profession, years of work were tested but they could not improve the performance of the models. The previously published literature had revealed none of the sociodemographic characteristics was associated with psychological stress, anxiety, and depression [38].

Each latent variable had three or more item variables, and the parameters in the associated linear equations of SOFAIDC were positive, consistent with the results of factor analysis. Meanwhile, some item variables were affected by one or more latent variables and the parameters were positive, such as *T3*, which was affected by latent variables *f1 and f2*.

Although the factors and relationships of FMWs’ stress have been known, the hospital protection measures should be precise and targeted. Thus, the stress trends of each FMW in the frontline clinics should be known.

### The decreasing trend of FMWs’ stress over week

In Table 4, the results of the paired T-test showed the t-test statistics for the stress self-rating score of the current week versus the previous week were all negative, and the p-values were less than 0.006, indicating that FMWs’ stress significantly decreases over weeks. The decreasing trend of FMWs’ stress over weeks was such that FMWs’ stress self-rating scores were the highest in the first week, and the stress self-rating score decreased thereafter. By the sixth week, the stress self-rating score is 42.637% of the first week’s stress self-rating score.

**Table 4:** Paired t-tests for FMWs’ stress self-rating scores

Period	Average of stress self-rating score	Paired sample T-test for stress self-rating score of current week via previous week					Average of new stress self-rating score	Average of cum stress self-rating score	Curve of time with decreasing
		Week2	Week3	Week4	Week5	Week6			
Week1	64.45 ± 31.64	−10.06^****^	−11.22^****^	−12.32^****^	−11.64^****^	−11.48^****^	64.45	64.45	100%
Week2	53.63 ± 29.70		−8.70^****^	−11.03^****^	−10.33^****^	−10.30^****^	−10.48	53.97	83.738%
Week3	44.56 ± 29.17			−7.88^****^	−7.43^****^	−8.13^****^	−9.05	44.92	69.695%
Week4	35.80 ± 28.42				−3.79^***^	−5.32^****^	−8.76	36.15	56.102%
Week5	30.80 ± 29.80					−2.82^****^	−5.12	31.03	48.156%
Week6	27.06 ± 30.33						−3.56	27.48	42.637%

***P < 0.01; ****P < 0.001

In Table 5, the parameter $\:{\text{f}}_{\text{a}\text{l}\text{p}\text{h}\text{a}}$ for LGM30 was 80.47, indicating FMWs had experienced relatively high stress during the first week in frontline clinics. The parameter $\:{\text{f}}_{\text{b}\text{e}\text{t}\text{a}}$ for LGM30 was − 14.2160, indicating the FMWs’ stress self-rating score could decrease as the number of weeks in frontline clinics increases. The parameter $\:{\text{f}}_{\text{g}\text{a}\text{m}\text{m}\text{a}}$ for LGM30 was 0.9045, which means the stress self-rating score could increase with the quadratic increase in the number of weeks.

**Table 5:** estimations of LGM30

wn	F_alpha_	f_beta_	f_gamma_	Error Randon effect	R-square
1 2 3 4 5 6	80.4694^****^	-14.2160^****^	0.9045^****^	13.4557*** 32.4252*** 32.4252^****^ 48.5612^****^ 159.1722^****^ 159.1722^****^	1 0.9842 0.9601 0.9576 0.8148

***P < 0.01; ****P < 0.001. *wn* the number of weeks in frontline clinics

The overall stress trends have been known, which can be used to decide when FMWs should be evacuated from the frontline clinics timely to avoid increasing their stress.

### Distinguishing and selection of low stress trend

Trajectories of stress trends for the 4-class model were depicted (see Supplemental Fig. 2). The A-class was a trajectory (15.2% of participants) characterized by a significant intercept ($\:{\upbeta\:}$= 26.4912, SE = 2.3265, *p* < 0.0001), but neither the linear nor quadratic slope was significant. The B-class was a trajectory (21.2% of participants), characterized by a significant intercept ($\:{\upbeta\:}$= 38.5755, SE = 6.5218, *p* < 0.0001) and a linear slope ($\:{\upbeta\:}$= −12.1153, SE = 1.8189, *p* < 0.0001). The C-class was a trajectory (42.1% of participants), characterized by a significant intercept ($\:{\upbeta\:}$= 98.3429, SE = 3.1255, *p* < 0.0001) and a linear slope ($\:{\upbeta\:}$= −14.5021, SE = 0.7902, *p* < 0.0001). Finally, the D-class was a trajectory (21.4% of participants), characterized by a significant intercept ($\:{\upbeta\:}$= 95.5991, SE = 3.9697, *p* < 0.0001) and a linear slope ($\:{\upbeta\:}$= −4.4729, SE = 1.0871, *p* < 0.0001).

In Supplemental Fig. 2, although stress trend B-class and C-class have low and high stress self-rating scores in the first week, they decline over weeks and reach low stress levels in the first six weeks. Hence, MWs with stress trend B-class or C-class should be prioritized for frontline clinics. MWs with stress trend D-class may remain high and slightly decrease during the frontline clinics, and should not be prioritized for frontline clinics. MWs with stress trend A-class have a lower level of stress, but the stress trend increases to varying degrees over weeks, and some of these MWs should not be prioritized for the frontline clinics. Therefore, all MWs with stress trend D-class and some MWs with stress trend A-class which experience an increase quickly over weeks were ‘high’ stress cases, accounting for 22.4% of the total, and the other MWs were ‘low’ stress cases.

The bin, WOE, and IV of all variables were displayed (see Supplemental Table 6). In Table 6, the sum of IV of working year, *T1*, *T8*, *T14*, *T15*, *T20*, and education level were 0.1226, 1.3954, 0.6707, 0.0201, 0.4360, 0.3780, and 0.233, respectively. All of these values were greater than 0.02, indicating that they can be used to build a stress scorecard to determine whether a MW’s stress trend will be a low stress trend when the MW will be working in frontline clinics.

**Table 6:** Stress scorecard for assessing whether or not a MW was low anxious

Variables	Upper limit	Lower limit	WOE	IV	Bin	No of Bin	Parameter	Scores
*Intercept*	-	-	-	-	-	-	−1.45282^***^	0
*Working year*	100	15	−0.26329	0.01322	3	22	1.20381^**^	76.431
*Working year*	15	10	0.54764	0.0866	2	27		48.264
*Working year*	10	0	−0.21147	0.02282	1	58		74.631
*T1*	4.0001	3.7	2.3394	1.05696	3	16	0.52155^***^	32.081
*T1*	3.1	2.8	−0.47401	0.10779	2	59		74.419
*T1*	2.2	0.9999	−1.0279	0.23066	1	32		82.754
*T8*	4.0001	2.8	1.24079	0.40425	3	22	0.29031^***^	56.892
*T8*	2.2	1.9	−0.08097	0.00228	2	38		67.964
*T8*	1.3	0.9999	−0.88744	0.26418	1	47		74.72
*T14*	4.0001	2.8	−0.00241	0	3	67	1.83276^***^	67.413
*T14*	2.2	1.9	0.10135	0.00326	2	33		61.926
*T14*	1.3	0.9999	−0.55097	0.01687	1	7		96.422
*T15*	4.0001	3.7	1.24079	0.33075	3	18	0.71368^***^	41.735
*T15*	3.1	1.9	−0.30514	0.05897	2	74		73.569
*T15*	1.3	0.9999	−0.63102	0.04625	1	15		80.28
*T20*	3.1	2.8	−0.2145	0.01497	2	37	0.47163^***^	70.205
*T20*	2.2	0.9999	−0.80691	0.16689	1	35		78.266
*T20*	4.0001	3.7	0.71469	0.19769	3	35		57.56
*Education level*	Post graduate		−1.46726	0.204	3	16	0.74022^***^	121.373
*Education level*	Junior college or below		0.3245	0.022	1	21		55.324
*Education level*	Undergraduate		0.10371	0.007	2			

**P < 0.05; ***P < 0.01

The parameters of working year, *T1*, *T8*, *T14*, *T15*, *T20*, and education level were 1.20381, 0.52155, 0.29031, 1.83276, 0.71368, 0.47163, and 0.74022, respectively. They were all significant, indicating that the influence of the independent variables in the stress scorecard on the dependent variable is significant.

Each variable has been binned and the WOE and scores can be calculated for each bin of each variable. When the value of the corresponding variable falls into the corresponding bin, it can be converted into a score, and then the accumulated score becomes the MW’s stress trend score, which can determine whether a MW’s stress trend will be a low-stress trend when the MW is working in frontline clinics.

In Supplemental Fig. 5, the result of stress scorecard loopback testing showed the lower the stress trend score, the higher the FMWs’ stress trend, which means the scorecard could be used to assess whether a MW was at low stress trend or not. The stress scorecard has been transformed into Excel, SQL formats (in Supplemental Material 1) for convenient use.

## Discussion

The objective of this study is to to alleviate the FMWs’ psychological stress due to COVID-19 by optimizing hospital protection measures for FMWs, which includes refining the selection process, improving protective measures for their work in frontline clinics, and determining the appropriate time for evacuation.

Selecting appropriate MWs is a crucial aspect of hospital protection. The overall stress self-rating score was the highest in the first week, with a subsequent decrease in stress trend, possibly because individuals had an acute reaction to unknown risk or danger and adapted to circumstance [7, 21, 23, 33]. However, this study showed that some FMWs had still experienced high levels of stress while working in the frontline clinics. The COVID-19 pandemic has caused a significant increase in stress for FMWs who had a much higher perceived stress level than average [24, 38, 53]. Hence, it is necessary to prefect this hospital protection.

The previous published literature gave four categories of anxiety trends [28, 42]. The stress trends also can be divided into four categories, and this study had given four categories of FMWs’ stress trends, which indicate some FMWs still experience high levels of stress while working in the frontline clinics. We need tools to distinguish whether the stress trend of MWs is of low type.

The stress scorecard was developed to distinguish whether or not the stress trend is a low stress tend and determine who has priority to work in frontline clinics. The data of education level and working year could be collected from the human resource or the personnel files. The data of *T1* (anxiety about being infected), *T8* (feeling of being avoided by others), *T14* (Elevated mood), *T15* (sleep disturbances), *T20* (Burden of being unable to take care of family members due to work) could be collected via communication or questionnaire, which could cover the contents. These data could be collected and translated into the stress tend score before the hospital management decide which MWs can enter the frontline clinics.

The stress scorecard could assist hospital managers in choosing the appropriate MWs to work at the frontline clinics, to achieve hospital protection improvement. The SQL codes of the stress scorecard were listed in appendix (in supplemental material 1) for easy reference and use by hospital managers.

The previous studies showed that FMWs were more stressful, but their job satisfaction was higher due to the improvements in organizational support [61, 65]. Adequate support needs to be given to the population to avoid increased stress [74, 80]. Support can reduce feelings of stress, emotional exhaustion, and increase feelings of personal accomplishment, which can lead to improvements in their mental health and job performance if one is experiencing issues with anxiety, depression, and mental well-being (Alan Maddock, 2023), [38]. Targeted, well-designed mental health interventions can improve outcomes among health care workers [90]. But the stress trends of different FMWs are different. Hence, the stress structure or factor should be explored and confirmed, and the relationships should be measured to find the actionable interventions to decrease FMWs’ stress severity.

EFA was applied to explore the factors of FMWs’ stress, and the factors were confirmed via SEM. Additionally, SEM revealed and measured the relationships between items of the Chinese version of stress-related questions and the factors. This study showed that hospital protections could purposefully reduce the FMWs’ stress through *Factor1*, *Factor2*, *Factor3*, and *Factor4* because the parameter between *T10* (Feeling of being protected by hospital) and *fg* was negative and significant, and the parameters between four factors and *fg* were positive. The total effect of the stress-related questions and factors from *T10* were negative (see Table 3) based on the SOFAIDC model. Hence, the improvement of hospital protection measures can enhance FMWs’ feeling of being protected by hospital to alleviate FMWs’ stress, indicating that the hospital protections can alleviate FMWs’ stress.

As the *Factor1*was concluded as ‘anxiety of infection’, it could be reduced by improving corresponding hospital protections based on SOFAIDC. FMWs’ anxiety plays critical roles in mitigating the detrimental effects of perceived stress on mental health [15], and inadequate protective equipment is associated with higher levels of stress and anxiety in FMWs [14, 68]. Healthcare professionals were most affected by protective measures at their workplace and changes in work procedures [95]. Hospitals could provide adequate protective equipment and other logistical support, establish and improve epidemic-proof medical workflows, create a working environment that meets epidemic-proof standards, and monitor biological security in real-time to perfect hospital protection and to reduce the ‘anxiety of infection’ .

Since the *Factor2*was concluded as ‘exhaustion’, it could be reduced via improving corresponding hospital protections based on SOFAIDC. Mindfulness, coping skills development, and relaxation and reflection interventions repeatedly influenced measures of burnout, stress, depression, and emotional exhaustion, result in the most significant changes in mental health outcomes [17, 55]. Having alternate rest periods was likely to reduce the risk of stress (Thu Pham H et al., 2023). Whether MWs can get compensatory leave and job satisfaction at work were the main factors affecting the work stress level of medical workers (He, G., et al., 2023). Hospitals could improve the rationality of FMWs schedules, provide compensatory leave for overtime and night shifts, encourage FMWs to practice Tai Chi, expressive writing, art therapy and yoga, etc., which are also associated with positive changes in mental health outcome measures [17] and ensure their nutrition, to enhance hospital protections and relieve the exhaustion of FMWs.

As the *Factor3*was concluded as ‘lack of cognition for infection’, it could be reduced through the improvement of corresponding hospital protections based on SOFAIDC. The cognitive appraisals can significantly influence perceived self-efficacy and psychological distress in prolonged stress situations like lockdowns [17]. Hospitals should provide training on epidemic prevention knowledge, medical and nursing workflow, and use of the working environment, interface with logistics before MWs transition to become FMWs. They could also arrange for experienced experts to provide guidance when FMWs will be working in the frontline clinics, to perfect hospital protections and enhance awareness of infection.

As the *Factor4*was concluded as ‘hesitant feeling’, it could be reduced by improving corresponding hospital protections based on SOFAIDC. Job satisfaction is higher due to the improvements in monetary compensation, reward [24, 65]. Hospitals should pay FMWs adequate monetary compensation, actively promote FMWs’ sense of responsibility, professional ethics and their safety, and arrange for professional psychological counselors to provide psychological encouragement and support to FMWs when they enter the frontline clinics each day. Additionally, to perfect hospital protections and ameliorate FMWs’ hesitant feeling, hospital management should actively communicate with the government to take care of FMW’s families and dispel public misconceptions about them.

In addition, hospitals should recruit personnel with higher education levels and provide opportunities for in-depth learning to reduce their psychological stress, exhaustion, and anxiety. However, this approach could increase the FMWs’ burden of being unable to take care of family members due to work. Therefore, a balanced approach to education level in recruitment is needed, avoiding extremes of too high nor too low. Particular attention should be paid to young people, with less work experience and better education, as they are the most susceptible to the psychosocial burden and leave the profession the most often [43].

The hospital protective measures for reducing FMWs’ stress are perfected not only through the selection of FMWs and protective measures for FMWs working in the frontline clinics, but also by deciding when each FMW should withdraw from the frontline clinics.

This study found that the overall stress trend would decrease, and the stress self-rating score at the 6th week was 42.637% of that score at the first week, but the stress trend might increase over the quadratic function of number of weeks. There was a significant interaction between comorbid anxiety disorder and the linear and quadratic effects of time [76, 79]. With continuing exposure to relevant stressors over time, an interplay between stressors, anxiety, and anxiety sensitivity is likely to lead to increased anxiety [34, 46, 50]. Therefore, hospital management should decide when FMWs should leave the frontline clinics to improve hospital protection and should arrange for FMWs to be withdrawn after 8 weeks of work, and, at the latest, by the 16th week, to reduce stress and prevent long-term distress for FMWs based on the stress trend model of LGM30.

## Limitations and future directions

The results of this study should be interpreted considering several limitations, with implications for future research. First, the study had a relatively small sample size. Power was sufficient for the statistical analyses used; increasing the sample size in future studies may expand the range of available estimation methods for SEM. Second, the stress self-ratings scores were self-reported which may not be accurate, future studies may measure psychological indicators such as blood pressure to obtain more accurate psychological stress rating data. Third, the findings of this study have not been verified in regions other than China. Future research could explore how the structure of psychological stress changes over time and across regions, and determine through experiments whether FMWs’ psychological stress increases quadratically with the number of weeks they worked in frontline clinics.

## Conclusions

This study sought to alleviate the FMWs’ psychological stress due to COVID-19 by optimizing hospital protection measures, which includes refining the selection process, improving protective measures for their work in frontline clinics, and determining the appropriate time for evacuation. Findings provide support for the efficacy of the stress-related questions as a tool for measuring the stress during the pandemic, which have been verified for reliability and validity. The factors of FMWs’ psychological stress were explored by EFA and confirmed by SEM, revealing that improved hospital protection measures can alleviate the FMWs’ psychological stress. A stress scorecard was developed to select FMWs with a low stress trend, and a latent growth model was used to determine when FMWs should withdraw. The hospital protection measures aimed at improving hospital management and reducing the FMWs’ psychological stress.

## Supplementary Information

Supplementary Material 1.

Supplementary Material 2.

Supplementary Material 3.

Supplementary Material 4.

Supplementary Material 5.

Supplementary Material 6.

Supplementary Material 7.

Supplementary Material 8.

Supplementary Material 9.

Supplementary Material 10.

Supplementary Material 11.

Supplementary Material 12.

## Data Availability

The data in this study would be made available on request.
