# Supplementary material for: Enhancing hospital protection measures reduces frontline medical workers’ stress during the pandemic
Source: BMC Psychol. 2024 Dec 3;12:716. doi: 10.1186/s40359-024-02185-8 (PMC11613736; doi:10.1186/s40359-024-02185-8)
Supplement: Supplementary file 1 — Supplementary Material 1. [file 40359_2024_2185_MOESM1_ESM.docx]

**Supplemental Material 1** SQL code of anxious scorecard

SELECT (

(CASE

WHEN education in ('Junior college or below') THEN (55.323847589)

WHEN education in (' Undergraduate ') THEN (63.462728509)

ELSE (121.37330528)

END)

/*********************************************/

/* Variable : t1 *****/

/*********************************************/

+ (CASE

WHEN t1 <= (2.2) THEN (82.754320864)

WHEN t1 > (2.8) AND t1 <=(3.1) THEN (74.419017931)

ELSE (32.080579728)

END)

/*********************************************/

/* Variable : t14 *****/

/*********************************************/

+ (CASE

WHEN t14 <= (1.3) THEN (96.422422772)

WHEN t14 > (1.9) AND t14 <=(2.2) THEN (61.925960237)

ELSE (67.412988205)

END)

/*********************************************/

/* Variable : t15 *****/

/*********************************************/

+ (CASE

WHEN t15 <= (1.3) THEN (80.279913581)

WHEN t15 > (1.9) AND t15 <=(3.1) THEN (73.569270066)

ELSE (41.734783619)

END)

/*********************************************/

/* Variable : t20 *****/

/*********************************************/

+ (CASE

WHEN t20 <= (2.2) THEN (78.266467171)

WHEN t20 > (2.8) AND t20 <=(3.1) THEN (70.204736195)

ELSE (57.559830364)

END)

/*********************************************/

/* Variable : t8 *****/

/*********************************************/

+ (CASE

WHEN t8 <= (1.3) THEN (74.719558931)

WHEN t8 > (1.9) AND t8 <=(2.2) THEN ( 67.96396643)

ELSE (56.892037028)

END)

/*********************************************/

/* Variable : work_year *****/

/*********************************************/

+ (CASE

WHEN work_year <= 10 THEN (74.630861957)

WHEN work_year > 10 AND work_year <=15 THEN (48.263733118)

ELSE (76.430979166)

END)

)

AS Score_Anxiety_FMWs
