# Supplementary material for: Enhancing hospital protection measures reduces frontline medical workers’ stress during the pandemic
Source: BMC Psychol. 2024 Dec 3;12:716. doi: 10.1186/s40359-024-02185-8 (PMC11613736; doi:10.1186/s40359-024-02185-8)
Supplement: Supplementary file 2 — Supplementary Material 2. [file 40359_2024_2185_MOESM2_ESM.docx]

**Supplemental Table 2** indices for assessment of different models

| SEM type  Indices for  Assessment of model | SEM for factor analysis | SEM for second-order factor analysis | SEM for second-order factor analysis and associations between factors and demographic characters | SEM for second-order factor analysis and association among factors, items and demographic characters (SOFAIDC) |
| --- | --- | --- | --- | --- |
| Chi-Square(degrees of freedom) | 1127.51(102) | 9.49(97) | 42.89(106) | 31.93(117) |
| Fit Function | 45.1004 | 0.3795 | 0.4045 | 0.3012 |
| Root Mean Square Residual (RMSR) | 0.5759 | 0.0528 | 0.0514 | **0.0422** |
| Standardized RMSR(SRMSR) | 0.5759 | 0.0528 | 0.0514 | **0.0422** |
| Goodness of Fit Index(GFI) | 0.3058 | **0.9946** | **0.9949** | **0.9963** |
| Adjusted GFI(AGFI) | 0.1463 | **0.9924** | **0.9926** | **0.9946** |
| RMSEA | 0.6578 | **0.0625** | **0.0618** | **0.0507** |
| Probability of Close Fit (PCF) | 0 | **0.9992** | **1.0000** | **1.0000** |
| Bentler Comparative Fit Index (CFI) | 0.2883 | **1.0000** | **1.0000** | **1.0000** |
| Bentler-Bonett Normed Fit Index(NFI) | 0.2777 | **0.9939** | **0.9943** | **0.9958** |
| Bentler-Bonett Non-normed Index(NNFI) | 0.1627 | **1.0751** | **1.0110** | **1.0148** |
| Bollen Normed Index Rho1 | 0.1502 | **0.9925** | **0.9927** | **0.9945** |
| Bollen Non-normed Index Delta2 | 0.2971 | **1.0598** | **1.0085** | **1.0113** |
| James et al. Parsimonious NFI(PNFI) | 0.2360 | **0.8034** | **0.7750** | **0.7615** |
| Parsimonious GFI (PGFI) | 0.3058 | **0.8040** | **0.7754** | **0.7619** |
