# Supplementary material for: Enhancing hospital protection measures reduces frontline medical workers’ stress during the pandemic
Source: BMC Psychol. 2024 Dec 3;12:716. doi: 10.1186/s40359-024-02185-8 (PMC11613736; doi:10.1186/s40359-024-02185-8)
Supplement: Supplementary file 3 — Supplementary Material 3. [file 40359_2024_2185_MOESM3_ESM.docx]

**Supplemental Table 4** estimations of LGM28

| wn | $f_{\mathrm{alpha}}$ | $f_{\mathrm{beta}}$ | $f_{\mathrm{gamma}}$ | $\beta_{1}$ | $\beta_{2}$ | Error  Randon effect | R-square |
| --- | --- | --- | --- | --- | --- | --- | --- |
| 1  2  3  4  5  6 | 78.43372^****^ | -11.84975^****^ | 0.56201^**^ | - | - | 0^****^  12.2990^**^  32.3119^***^  32.3119^***^  154.5829^***^  154.5829^***^ | 0.9585  0.9801  0.9477  0.9413  0.8910  0.9558 |
|  |  |  |  | -0.0195 | -0.2189 |  |  |

**P*  0.10; ***P*  0.05;****P*  0.01; *****P*  0.001. wn: the number of weeks in frontline clinics.
