# Supplementary material for: Enhancing hospital protection measures reduces frontline medical workers’ stress during the pandemic
Source: BMC Psychol. 2024 Dec 3;12:716. doi: 10.1186/s40359-024-02185-8 (PMC11613736; doi:10.1186/s40359-024-02185-8)
Supplement: Supplementary file 4 — Supplementary Material 4. [file 40359_2024_2185_MOESM4_ESM.docx]

**Supplemental Table 5** statistics of trajectory analysis

| Trajectory Models | BIC | AIC | L | Bayes Factor |
| --- | --- | --- | --- | --- |
| 1 class-CNORM | -2108.6 | -2104.71 | -2101.71 |  |
| 2 class-CNORM | -1989.07 | -1981.28 | -1975.28 | 239.06 |
| 3 class-CNORM | -1983.65 | -1973.27 | -1965.27 | 10.84 |
| 4 class-CNORM | -1923.7 | -1909.43 | -1898.43 | 119.9 |
| 5 class-CNORM | -1928.3 | -1911.4 | -1898.4 | -9.2 |
| 6 class-CNORM | -1884.23 | -1864.77 | -1849.77 | 88.14 |
| 7 class-CNORM | -1867.6 | -1844.2 | -1826.2 | 33.28 |
