# Supplementary material for: Enhancing hospital protection measures reduces frontline medical workers’ stress during the pandemic
Source: BMC Psychol. 2024 Dec 3;12:716. doi: 10.1186/s40359-024-02185-8 (PMC11613736; doi:10.1186/s40359-024-02185-8)
Supplement: Supplementary file 5 — Supplementary Material 5. [file 40359_2024_2185_MOESM5_ESM.docx]

**Supplemental Table 6** WOE and IV of all stress-related variables

| **Variable Name** | **UL** | **LL** | **WOE** | **IV** | **Bin** | **Total** |
| --- | --- | --- | --- | --- | --- | --- |
| age | 9 | 7.25 | 0 | 0 | 3 | 2 |
| age | 7.25 | 3.75 | 0.07318 | 0.00301 | 2 | 59 |
| age | 3.75 | 2 | -0.04015 | 0.00069 | 1 | 46 |
| education | 4 | 3.7 | -1.46726 | 0.20403 | 3 | 16 |
| education | 2.2 | 1 | 0.3245 | 0.02248 | 1 | 21 |
| education | 3.1 | 2.8 | 0.10371 | 0.00724 | 2 | 70 |
| gender | 2 | 1.9 | 0.06915 | 0.00424 | 2 | 93 |
| gender | 1.1 | 1 | -0.55097 | 0.03374 | 1 | 14 |
| prof | 1.1 | 1 | -1.5318 | 0.23146 | 1 | 17 |
| prof | 2 | 1.9 | 0.17159 | 0.02593 | 2 | 90 |
| submit_t | 41 | 34.75 | -1.01051 | 0.14711 | 3 | 21 |
| submit_t | 34.75 | 22.25 | 0.54764 | 0.00962 | 2 | 3 |
| submit_t | 22.25 | 16 | 0.15817 | 0.02025 | 1 | 83 |
| t1 | 2.2 | 1 | -1.0279 | 0.23066 | 1 | 32 |
| t1 | 4 | 3.7 | 2.3394 | 1.05696 | 3 | 16 |
| t1 | 3.1 | 2.8 | -0.47401 | 0.10779 | 2 | 59 |
| t10 | 4 | 3.7 | -0.49968 | 0.13495 | 3 | 67 |
| t10 | 3.1 | 2.8 | 0.45233 | 0.06858 | 2 | 32 |
| t10 | 2.2 | 1 | 1.24079 | 0.147 | 1 | 8 |
| t11 | 4 | 3.7 | 0.98947 | 0.1813 | 3 | 16 |
| t11 | 3.1 | 1.9 | -0.0714 | 0.00308 | 2 | 66 |
| t11 | 1.3 | 1 | -0.75164 | 0.10528 | 1 | 25 |
| t12 | 4 | 2.8 | 1.52847 | 0.399 | 3 | 14 |
| t12 | 2.2 | 1.9 | -0.04015 | 0.00069 | 2 | 46 |
| t12 | 1.3 | 1 | -0.68103 | 0.16615 | 1 | 47 |
| t13 | 4 | 2.8 | 1.24079 | 0.40425 | 3 | 22 |
| t13 | 2.2 | 1.9 | 0.30252 | 0.02961 | 2 | 32 |
| t13 | 1.3 | 1 | -1.26474 | 0.53586 | 1 | 53 |
| t14 | 2.2 | 1.9 | 0.10135 | 0.00326 | 2 | 33 |
| t14 | 1.3 | 1 | -0.55097 | 0.01687 | 1 | 7 |
| t14 | 4 | 2.8 | -0.00241 | 0 | 3 | 67 |
| t15 | 4 | 3.7 | 1.24079 | 0.33075 | 3 | 18 |
| t15 | 3.1 | 1.9 | -0.30514 | 0.05897 | 2 | 74 |
| t15 | 1.3 | 1 | -0.63102 | 0.04625 | 1 | 15 |
| t16 | 4 | 3.7 | 0.59893 | 0.11245 | 2 | 29 |
| t16 | 3.1 | 1 | -0.27904 | 0.05239 | 1 | 78 |
| t17 | 4 | 3.7 | 1.16074 | 0.39857 | 3 | 25 |
| t17 | 3.1 | 2.8 | -0.12019 | 0.0064 | 2 | 49 |
| t17 | 2.2 | 1 | -1.50005 | 0.43526 | 1 | 33 |
| t18 | 4 | 3.7 | -0.07489 | 0.00267 | 3 | 52 |
| t18 | 3.1 | 2.8 | 0.11478 | 0.00622 | 2 | 49 |
| t18 | 2.2 | 1 | -0.36865 | 0.00685 | 1 | 6 |
| t19 | 4 | 3.7 | -0.36865 | 0.01369 | 3 | 12 |
| t19 | 3.1 | 2.8 | 1.14548 | 0.32547 | 2 | 21 |
| t19 | 2.2 | 1 | -0.40144 | 0.09915 | 1 | 74 |
| t2 | 4 | 3.7 | 1.44146 | 0.50437 | 3 | 20 |
| t2 | 3.1 | 1.9 | -0.41744 | 0.10813 | 2 | 75 |
| t2 | 1.3 | 1 | -1.15711 | 0.10514 | 1 | 12 |
| t20 | 3.1 | 2.8 | -0.2145 | 0.01497 | 2 | 37 |
| t20 | 2.2 | 1 | -0.80691 | 0.16689 | 1 | 35 |
| t20 | 4 | 3.7 | 0.71469 | 0.19769 | 3 | 35 |
| t22 | -20.25 | -72.75 | 0.02439 | 0.00059 | 3 | 105 |
| t22 | -72.75 | -99 | 0 | 0 | 1 | 2 |
| t3 | 4 | 3.7 | 1.24079 | 0.3675 | 3 | 20 |
| t3 | 2.2 | 1 | -1.12634 | 0.29346 | 1 | 35 |
| t3 | 3.1 | 2.8 | -0.07489 | 0.00267 | 2 | 52 |
| t30 | 0.1 | 0 | 0.10135 | 0.00326 | 1 | 33 |
| t30 | 1 | 0.9 | -0.04707 | 0.00151 | 2 | 74 |
| t31 | 1 | 0.9 | 0.05702 | 0.00197 | 2 | 64 |
| t31 | 0.1 | 0 | -0.08835 | 0.00306 | 1 | 43 |
| t32 | 1 | 0.9 | 0.48701 | 0.06259 | 2 | 25 |
| t32 | 0.1 | 0 | -0.17628 | 0.02265 | 1 | 82 |
| t33 | 1 | 0.9 | 0.22919 | 0.00782 | 2 | 15 |
| t33 | 0.1 | 0 | -0.04015 | 0.00137 | 1 | 92 |
| t34 | 0.1 | 0 | -0.49381 | 0.07883 | 1 | 40 |
| t34 | 1 | 0.9 | 0.23934 | 0.03821 | 2 | 67 |
| t35 | 1 | 0.9 | 0.03681 | 0.00017 | 2 | 13 |
| t35 | 0.1 | 0 | -0.00515 | 0.00002 | 1 | 94 |
| t4 | 4 | 3.7 | 0.6048 | 0.10292 | 3 | 26 |
| t4 | 3.1 | 2.8 | 0.30923 | 0.04455 | 2 | 46 |
| t4 | 2.2 | 1 | -1.56257 | 0.49105 | 1 | 35 |
| t5 | 4 | 3.7 | 1.8004 | 0.43835 | 3 | 11 |
| t5 | 3.1 | 2.8 | 0.06213 | 0.00187 | 2 | 51 |
| t5 | 2.2 | 1 | -0.83865 | 0.22945 | 1 | 45 |
| t6 | 4 | 2.8 | 1.24079 | 0.3675 | 3 | 20 |
| t6 | 2.2 | 1.9 | 0.01212 | 0.00007 | 2 | 53 |
| t6 | 1.3 | 1 | -1.5318 | 0.46292 | 1 | 34 |
| t7 | 4 | 2.8 | 0.90431 | 0.22426 | 3 | 24 |
| t7 | 2.2 | 1.9 | 0.06807 | 0.00243 | 2 | 55 |
| t7 | 1.3 | 1 | -2.05505 | 0.58288 | 1 | 28 |
| t8 | 1.3 | 1 | -0.88744 | 0.26418 | 1 | 47 |
| t8 | 4 | 2.8 | 1.24079 | 0.40425 | 3 | 22 |
| t8 | 2.2 | 1.9 | -0.08097 | 0.00228 | 2 | 38 |
| t9 | 4 | 3.7 | -0.64194 | 0.16854 | 2 | 53 |
| t9 | 3.1 | 1 | 0.46308 | 0.12158 | 1 | 54 |
| use_T | 715 | 557.75 | 0.54764 | 0.01924 | 3 | 6 |
| use_T | 557.75 | 243.25 | -0.70512 | 0.12035 | 2 | 32 |
| use_T | 243.25 | 86 | 0.19933 | 0.02702 | 1 | 69 |
| work_year | 5 | 3.8 | -0.26329 | 0.01322 | 3 | 22 |
| work_year | 3 | 2.6 | 0.54764 | 0.0866 | 2 | 27 |
| work_year | 2.2 | 1 | -0.21147 | 0.02282 | 1 | 58 |
