# Supplementary material for: Enhancing hospital protection measures reduces frontline medical workers’ stress during the pandemic
Source: BMC Psychol. 2024 Dec 3;12:716. doi: 10.1186/s40359-024-02185-8 (PMC11613736; doi:10.1186/s40359-024-02185-8)
Supplement: Supplementary file 6 — Supplementary Material 6. [file 40359_2024_2185_MOESM6_ESM.docx]

**Supplemental Table 3** indices for assessment of different latent growth curve models

| Model Type | AIC | BCAIC | SBC | SRMSR | AGFI | RMSEA | PCF | NNFI | PNFI | PGFI |
| --- | --- | --- | --- | --- | --- | --- | --- | --- | --- | --- |
| LGM1 | 381.51 | 311.15 | 335.15 | 0.2786 | 0.3252 | 0.5813 | 0.0000 | 0.1460 | 0.2333 | **0.6402** |
| LGM2 | 293.46 | 237.75 | 256.75 | 0.3913 | 0.2309 | 0.5735 | 0.0000 | 0.3410 | 0.4319 | **0.5811** |
| LGM3 | 72.64 | 11.07 | 32.07 | 0.0672 | 0.7171 | 0.2986 | 0.0000 | 0.7721 | **1.0809** | **1.0919** |
| LGM4 | 24.70 | -25.14 | -8.14 | 0.0970 | 0.7238 | 0.2215 | 0.0000 | 0.8833 | **1.0011** | **0.9362** |
| LGM5 | 380.99 | 313.56 | 336.56 | 0.2892 | 0.2745 | 0.5927 | 0.0000 | 0.1510 | 0.2316 | **0.5858** |
| LGM6 | 72.13 | 19.36 | 37.36 | 2.0856 | 0.5276 | 0.3165 | 0.0000 | 0.7856 | **0.9420** | **0.8221** |
| LGM7 | 73.19 | 32.14 | 46.14 | 1.7231 | 0.4488 | 0.3529 | 0.0000 | 0.7988 | **0.7456** | **0.6666** |
| LGM8 | 143.25 | 78.75 | 100.75 | 0.4718 | 0.6631 | 0.3876 | 0.0000 | 0.6277 | **0.9206** | **1.0640** |
| LGM9 | 103.01 | 50.24 | 68.24 | 0.5610 | 0.6225 | 0.3667 | 0.0000 | 0.7236 | **0.8683** | **0.8980** |
| LGM10 | 73.03 | 14.67 | 34.67 | 0.0694 | 0.6949 | 0.3055 | 0.0000 | 0.7747 | **1.0330** | **1.0320** |
| LGM11 | 70.73 | 23.83 | 39.83 | 1.6282 | 0.5242 | 0.3293 | 0.0000 | 0.7957 | **0.8488** | **0.7659** |
| LGM12 | 80.50 | 33.59 | 49.50 | 0.0714 | 0.6267 | 0.3473 | 0.0000 | 0.7763 | **0.8281** | **0.8307** |
| LGM13 | 76.74 | 44.49 | 55.49 | 1.8610 | 0.3286 | 0.3994 | 0.0000 | 0.8037 | **0.5894** | **0.5327** |
| LGM14 | 110.91 | 52.27 | 72.27 | 0.0939 | 0.6439 | 0.3618 | 0.0000 | 0.6999 | **0.9333** | **0.9816** |
| LGM15 | 73.52 | 29.55 | 44.55 | 1.6115 | 0.4926 | 0.3436 | 0.0000 | 0.7942 | **0.7942** | **0.7181** |
| LGM16 | 109.03 | 62.12 | 78.12 | 0.0946 | 0.5200 | 0.3953 | 0.0000 | 0.7196 | **0.7676** | **0.7630** |
| LGM17 | 75.94 | 43.69 | 54.69 | 1.7644 | 0.3416 | 0.3976 | 0.0000 | 0.8053 | **0.5905** | **0.5366** |
| LGM18 | 50.20 | 0.36 | 17.36 | **0.0291** | 0.7028 | 0.2812 | 0.0000 | 0.8326 | **0.9436** | **0.9213** |
| LGM19 | -6.49 | -44.61 | -31.61 | **0.0237** | 0.8710 | 0.1001 | **0.1834** | **0.9612** | **0.8331** | **0.8128** |
| LGM20 | 51.65 | 4.74 | 20.74 | **0.0267** | 0.6829 | 0.2310 | 0.0000 | 0.8337 | **0.8893** | **0.8662** |
| LGM21 | -6.74 | -41.92 | -29.92 | **0.0184** | 0.8638 | 0.0936 | **0.2226** | **0.9657** | **0.7725** | **0.7516** |
| LGM22 | 40.37 | 5.19 | 17.19 | **0.0374** | 0.6842 | 0.2954 | 0.0000 | 0.8720 | **0.6976** | **0.6877** |
| LGM23 | 22.07 | -0.70 | 7.30 | 0.4701 | 0.6296 | 0.2723 | 0.0000 | **0.9230** | 0.4922 | 0.4748 |
| LGM24 | 47.92 | -13.65 | 7.35 | 0.0540 | 0.8848 | 0.2562 | 0.0000 | 0.8237 | **0.8237** | **0.9309** |
| LGM25 | -9.96 | **-59.80** | **-42.80** | **0.0461** | **0.9491** | 0.0910 | **0.2094** | **0.9529** | **0.7714** | **0.7896** |
| LGM26 | 35.48 | -17.29 | 0.71 | 0.0526 | 0.8997 | 0.2438 | 0.0000 | 0.8598 | **0.7370** | **0.8129** |
| LGM27 | -16.25 | -57.30 | -43.30 | **0.0490** | **0.9750** | **0.0000** | **0.7360** | **0.9770** | **0.6513** | **0.6601** |
| LGM28 | -15.17 | **-73.81** | **-53.81** | **0.0453** | **0.9552** | **0.0695** | **0.3367** | **0.9513** | **0.9060** | **0.9280** |
| LGM29 | -10.12 | -51.18 | -37.18 | **0.0183** | 0.8746 | **0.0743** | **0.3206** | **0.9645** | **0.9002** | **0.8746** |
| LGM30 | -11.39 | **-58.30** | **-42.30** | **0.0381** | 0.8906 | **0.0759** | **0.3052** | **0.9590** | **1.0230** | **0.9975** |
| LGM31 | 360.34 | 295.84 | 317.84 | 0.3375 | 0.2694 | 0.5896 | 0.0000 | 0.1961 | 0.2876 | 0.5935 |

LGM1: $\mathrm{score}_{\mathrm{wn}}=f_{\mathrm{alpha}} +\varepsilon_{1}$;

LGM2: $\mathrm{score}_{\mathrm{wn}}=f_{\mathrm{alpha}} +\varepsilon_{1}$, $\varepsilon_{1}$ being with random effect;

LGM3: $\mathrm{score}_{\mathrm{wn}}=f_{\mathrm{alpha}} + f_{\mathrm{beta}}\times wn+\varepsilon_{1}$;

LGM4: $\mathrm{score}_{\mathrm{wn}}=f_{\mathrm{alpha}} + f_{\mathrm{beta}}\times wn+\varepsilon_{1}$,$\varepsilon_{1}$ being with random effect;

LGM5: $\mathrm{score}_{\mathrm{wn}}=f_{\mathrm{alpha}} +\beta_{1}\times\mathrm{score}_{\mathrm{wn}-1}+\varepsilon_{1}$;

LGM6: $\mathrm{score}_{\mathrm{wn}}=f_{\mathrm{alpha}} +\beta_{1}\times\mathrm{score}_{\mathrm{wn}-1}+\varepsilon_{1}$,$\varepsilon_{1}$ being with random effect;

LGM7: $\mathrm{score}_{\mathrm{wn}}=f_{\mathrm{alpha}} +\beta_{1}\times\mathrm{score}_{\mathrm{wn}-1}+\varepsilon_{1}$,$\beta_{1}\mathrm{and}\varepsilon_{1} being with random effect;$

LGM8: $\mathrm{score}_{\mathrm{wn}}=f_{\mathrm{alpha}}+f_{\mathrm{gamma}}\times\mathrm{wn}^{2} +\varepsilon_{1}$;

LGM9: $\mathrm{score}_{\mathrm{wn}}=f_{\mathrm{alpha}}+f_{\mathrm{gamma}}\times\mathrm{wn}^{2} +\varepsilon_{1}$,$\varepsilon_{1}$ being with random effect;

LGM10: $\mathrm{score}_{\mathrm{wn}}=f_{\mathrm{alpha}}+\beta_{1}\times\mathrm{score}_{\mathrm{wn}-1}+ f_{\mathrm{beta}}\times wn +\varepsilon_{1}$;

LGM11: $\mathrm{score}_{\mathrm{wn}}=f_{\mathrm{alpha}}+\beta_{1}\times\mathrm{score}_{\mathrm{wn}-1}+ f_{\mathrm{beta}}\times wn +\varepsilon_{1}$, $\varepsilon_{1}$ being with random effect;

LGM12: $\mathrm{score}_{\mathrm{wn}}=f_{\mathrm{alpha}}+\beta_{1}\times\mathrm{score}_{\mathrm{wn}-1}+ f_{\mathrm{beta}}\times wn +\varepsilon_{1}$, $\beta_{1}$ being with random effect;

LGM13: $\mathrm{score}_{\mathrm{wn}}=f_{\mathrm{alpha}}+\beta_{1}\times\mathrm{score}_{\mathrm{wn}-1}+ f_{\mathrm{beta}}\times wn +\varepsilon_{1}$, $\beta_{1}$ and $\varepsilon_{1}$ being with random effect;

LGM14: $\mathrm{score}_{\mathrm{wn}}=f_{\mathrm{alpha}}+\beta_{1}\times\mathrm{score}_{\mathrm{wn}-1}+f_{\mathrm{gamma}}\times\mathrm{wn}^{2} +\varepsilon_{1}$;

LGM15: $\mathrm{score}_{\mathrm{wn}}=f_{\mathrm{alpha}}+\beta_{1}\times\mathrm{score}_{\mathrm{wn}-1}+f_{\mathrm{gamma}}\times\mathrm{wn}^{2} +\varepsilon_{1}$, $\varepsilon_{1}$ being with random effect;

LGM16: $\mathrm{score}_{\mathrm{wn}}=f_{\mathrm{alpha}}+\beta_{1}\times\mathrm{score}_{\mathrm{wn}-1}+f_{\mathrm{gamma}}\times\mathrm{wn}^{2} +\varepsilon_{1}$, $\beta_{1}$ being with random effect;

LGM17: $\mathrm{score}_{\mathrm{wn}}=f_{\mathrm{alpha}}+\beta_{1}\times\mathrm{score}_{\mathrm{wn}-1}+f_{\mathrm{gamma}}\times\mathrm{wn}^{2} +\varepsilon_{1}$, $\beta_{1}$ and $\varepsilon_{1}$ being with random effect;

LGM18: $\mathrm{score}_{\mathrm{wn}}=f_{\mathrm{alpha}}+ f_{\mathrm{beta}}\times wn+f_{\mathrm{gamma}}\times\mathrm{wn}^{2} +\varepsilon_{1}$;

LGM19: $\mathrm{score}_{\mathrm{wn}}=f_{\mathrm{alpha}}+ f_{\mathrm{beta}}\times wn+f_{\mathrm{gamma}}\times\mathrm{wn}^{2} +\varepsilon_{1}$, $\varepsilon_{1}$ being with random effect;

LGM20: $\mathrm{score}_{\mathrm{wn}}=f_{\mathrm{alpha}}+\beta_{1}\times\mathrm{score}_{\mathrm{wn}-1}+ f_{\mathrm{beta}}\times wn+f_{\mathrm{gamma}}\times\mathrm{wn}^{2} +\varepsilon_{1}$;

LGM21: $\mathrm{score}_{\mathrm{wn}}=f_{\mathrm{alpha}}+\beta_{1}\times\mathrm{score}_{\mathrm{wn}-1}+ f_{\mathrm{beta}}\times wn+f_{\mathrm{gamma}}\times\mathrm{wn}^{2} +\varepsilon_{1}$, $\varepsilon_{1}$ being with random effect;

LGM22: $\mathrm{score}_{\mathrm{wn}}=f_{\mathrm{alpha}}+\beta_{1}\times\mathrm{score}_{\mathrm{wn}-1}+ f_{\mathrm{beta}}\times wn+f_{\mathrm{gamma}}\times\mathrm{wn}^{2} +\varepsilon_{1}$, $\beta_{1}$ being with random effect;

LGM23: $\mathrm{score}_{\mathrm{wn}}=f_{\mathrm{alpha}}+\beta_{1}\times\mathrm{score}_{\mathrm{wn}-1}+ f_{\mathrm{beta}}\times wn+f_{\mathrm{gamma}}\times\mathrm{wn}^{2} +\varepsilon_{1}$, $\beta_{1}$ and $\varepsilon_{1}$ being with random effect;

LGM24: $\mathrm{score}_{\mathrm{wn}}=f_{\mathrm{alpha}}+\beta_{1}\times\mathrm{score}_{\mathrm{wn}-1}+ f_{\mathrm{beta}}\times wn+f_{\mathrm{gamma}}\times\mathrm{wn}^{2}+\beta_{2}\times education+\varepsilon_{1}$;

LGM25: $\mathrm{score}_{\mathrm{wn}}=f_{\mathrm{alpha}}+\beta_{1}\times\mathrm{score}_{\mathrm{wn}-1}+ f_{\mathrm{beta}}\times wn+f_{\mathrm{gamma}}\times\mathrm{wn}^{2}+\beta_{2}\times education+\varepsilon_{1}$, $\varepsilon_{1}$ being with random effect;

LGM26: $\mathrm{score}_{\mathrm{wn}}=f_{\mathrm{alpha}}+\beta_{1}\times\mathrm{score}_{\mathrm{wn}-1}+ f_{\mathrm{beta}}\times wn+f_{\mathrm{gamma}}\times\mathrm{wn}^{2}+\beta_{2}\times education+\varepsilon_{1}$, $\beta_{1}$ being with random effect;

LGM27: $\mathrm{score}_{\mathrm{wn}}=f_{\mathrm{alpha}}+\beta_{1}\times\mathrm{score}_{\mathrm{wn}-1}+ f_{\mathrm{beta}}\times wn+f_{\mathrm{gamma}}\times\mathrm{wn}^{2}+\beta_{2}\times education+\varepsilon_{1}$, $\beta_{1}$ and $\varepsilon_{1}$ being with random effect;

LGM28: $\mathrm{score}_{\mathrm{wn}}=f_{\mathrm{alpha}}+\beta_{1}\times\mathrm{score}_{\mathrm{wn}-1}+ f_{\mathrm{beta}}\times wn+f_{\mathrm{gamma}}\times\mathrm{wn}^{2}+\beta_{2}\times education+\varepsilon_{1}$, $\varepsilon_{1}$ being with random effect and ${\sigma^{2}(\varepsilon}_{1,wn-1})\leq{\sigma^{2}(\varepsilon}_{1,wn})$;

LGM29: $\mathrm{score}_{\mathrm{wn}}=f_{\mathrm{alpha}}+\beta_{1}\times\mathrm{score}_{\mathrm{wn}-1}+ f_{\mathrm{beta}}\times wn+f_{\mathrm{gamma}}\times\mathrm{wn}^{2} +\varepsilon_{1}$, $\varepsilon_{1}$ being with random effect and ${\sigma^{2}(\varepsilon}_{1,wn-1})\leq{\sigma^{2}(\varepsilon}_{1,wn})$

LGM30: $\mathrm{score}_{\mathrm{wn}}=f_{\mathrm{alpha}}+ f_{\mathrm{beta}}\times wn+f_{\mathrm{gamma}}\times\mathrm{wn}^{2} +\varepsilon_{1}$, $\varepsilon_{1}$ being with random effect and ${\sigma^{2}(\varepsilon}_{1,wn-1})\leq{\sigma^{2}(\varepsilon}_{1,wn})$;

LGM31: $\mathrm{score}_{\mathrm{wn}}=f_{\mathrm{alpha}} + f_{\mathrm{beta}}\times wn+\varepsilon_{1}$,$\varepsilon_{1}$ being with random effect and ${\sigma^{2}(\varepsilon}_{1,wn-1})\leq{\sigma^{2}(\varepsilon}_{1,wn})$

wn: the number of weeks in frontline clinics.
