# Supplementary material for: Enhancing hospital protection measures reduces frontline medical workers’ stress during the pandemic
Source: BMC Psychol. 2024 Dec 3;12:716. doi: 10.1186/s40359-024-02185-8 (PMC11613736; doi:10.1186/s40359-024-02185-8)
Supplement: Supplementary file 7 — Supplementary Material 7. [file 40359_2024_2185_MOESM7_ESM.docx]

**Supplemental Table 1** Factor analysis of the 20 stress-related questions

| **Factors** | **Questions** | **F1** | **F2** | **F3** | **F4** | **KMO'test** |
| --- | --- | --- | --- | --- | --- | --- |
| **Factor1:**  **Anxiety of infection**  **(cronbach'** $\boldsymbol{\alpha}$**=0.8181)** | *T1* | ***0.80*** | 0.27 | 0.20 | -0.21 | 0.83 |
|  | *T2* | ***0.62*** | 0.11 | 0.15 | -0.34 | 0.86 |
|  | *T5* | ***0.60*** | 0.34 | 0.31 | -0.21 | 0.89 |
| **Factor2:**  **exhaustion**  **(cronbach'** $\boldsymbol{\alpha}$**=0.8250)** | *T3* | 0.29 | ***0.53*** | 0.22 | -0.21 | 0.86 |
|  | *T4* | 0.21 | ***0.60*** | 0.30 | -0.10 | 0.89 |
|  | *T15* | -0.03 | ***0.46*** | 0.08 | -0.17 | 0.84 |
|  | *T16* | 0.04 | ***0.87*** | 0.07 | -0.13 | 0.84 |
|  | *T17* | 0.13 | ***0.81*** | 0.08 | -0.21 | 0.85 |
|  | *T20* | 0.26 | ***0.43*** | 0.19 | -0.27 | 0.93 |
| **Factor3:**  **Lack of cognition for infection**  **(cronbach'** $\boldsymbol{\alpha}$**=0.7582)** | *T6* | 0.14 | 0.17 | ***0.96*** | -0.18 | 0.79 |
|  | *T7* | 0.18 | 0.27 | ***0.80*** | -0.13 | 0.79 |
|  | *T8* | 0.20 | 0.30 | ***0.36*** | -0.32 | 0.92 |
|  | *T19* | 0.09 | 0.18 | ***0.39*** | -0.25 | 0.88 |
| **Factor4_negative:**  **hesitant feeling**  **(cronbach'** $\boldsymbol{\alpha}$**=0.767)** | *T11* | 0.19 | 0.31 | 0.36 | ***-0.39*** | 0.90 |
|  | *T12* | 0.22 | 0.28 | 0.40 | ***-0.47*** | 0.91 |
|  | *T13* | 0.21 | 0.34 | 0.20 | ***-0.48*** | 0.91 |
| **Factor_positive:**  **Protection incentive**  **(cronbach'** $\boldsymbol{\alpha}$**=0.663)** | *T9* | -0.04 | -0.12 | 0.00 | ***0.71*** | ***0.67*** |
|  | *T10* | -0.02 | -0.15 | -0.14 | ***0.77*** | ***0.77*** |
|  | *T14* | -0.17 | -0.07 | -0.08 | ***0.32*** | ***0.70*** |
|  | *T18* | -0.09 | -0.19 | -0.08 | ***0.38*** | ***0.71*** |
| **Unweighted variance explained by each factor** | | 3.22 | 2.58 | 2.56 | 1.85 |  |
| **Variance explained** | | 31.51% | 25.27% | 25.10% | 18.12% |  |
| **Kaiser-Meyer-Oklin 's Measure of Sampling Adequacy: Overall MSA** | | 0.8453 | | | |  |
| **Bartlett's test of sphericity** | | 1068.84(df=190, p<0.0001) | | | |  |
| **Correlation between factors** | |  |  |  |  |  |
|  | F2 | 0.0257 |  |  |  |  |
|  | F3 | 0.0006 | 0.0465 |  |  |  |
|  | F4 | 0.0282 | -0.0488 | -0.0477 |  |  |
