# Supplementary figures and images for: Enhancing hospital protection measures reduces frontline medical workers’ stress during the pandemic
Source: BMC Psychol. 2024 Dec 3;12:716. doi: 10.1186/s40359-024-02185-8 (PMC11613736; doi:10.1186/s40359-024-02185-8)

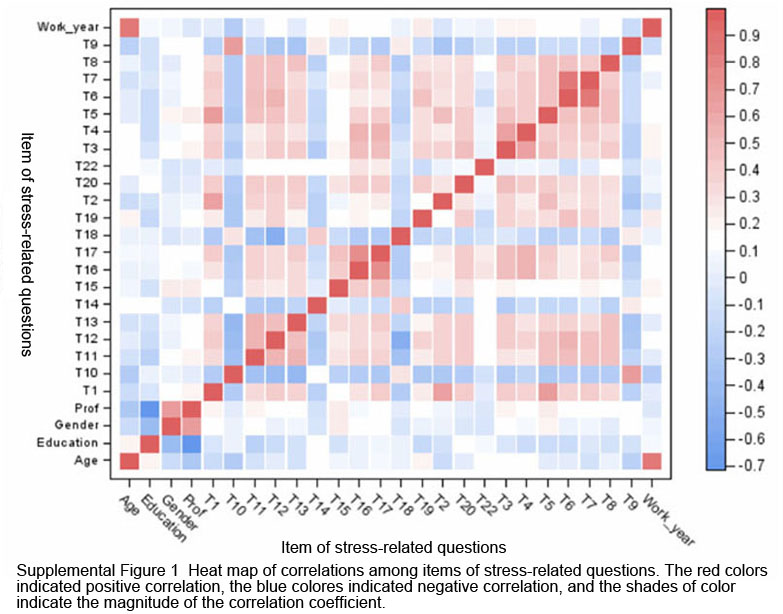

Supplement: Supplementary file 8 — Supplementary Material 8. [file 40359_2024_2185_MOESM8_ESM.jpg]

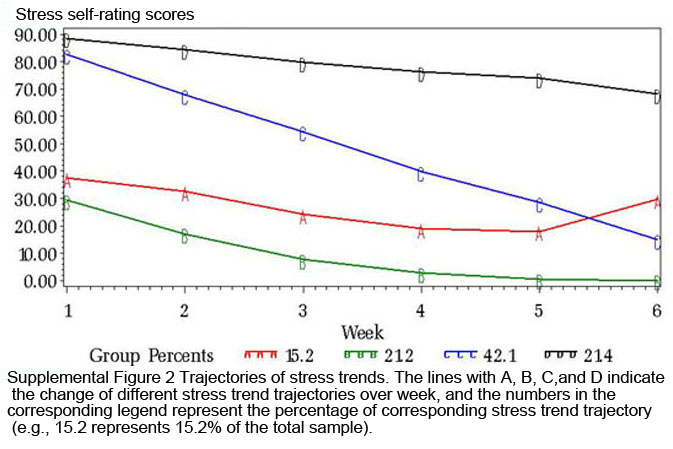

Supplement: Supplementary file 9 — Supplementary Material 9. [file 40359_2024_2185_MOESM9_ESM.jpg]

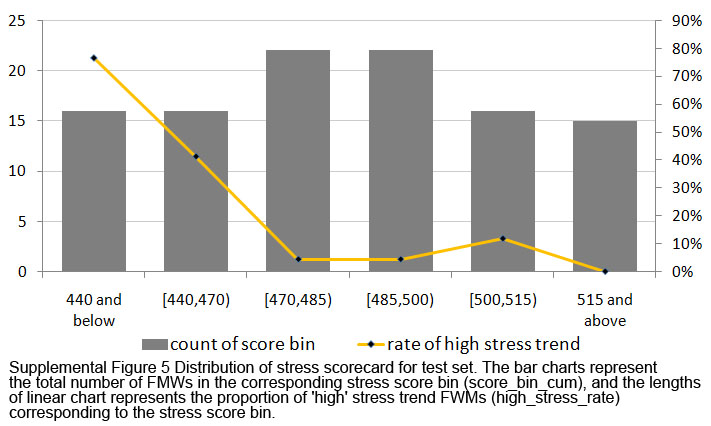

Supplement: Supplementary file 10 — Supplementary Material 10. [file 40359_2024_2185_MOESM10_ESM.jpg]

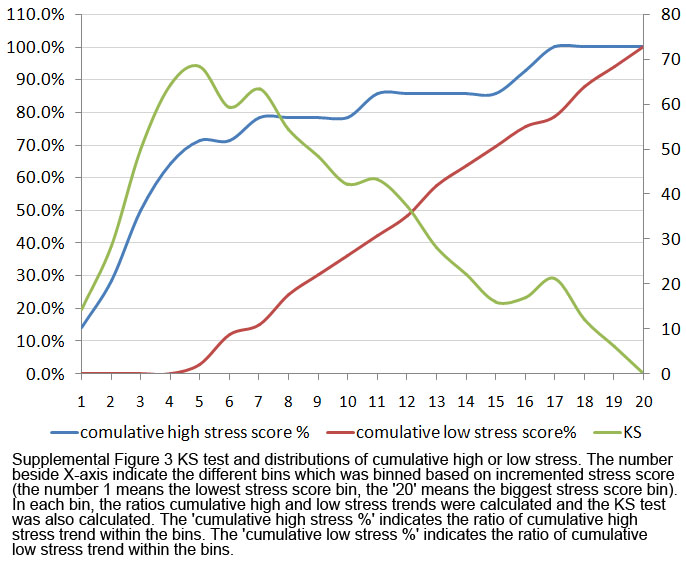

Supplement: Supplementary file 11 — Supplementary Material 11. [file 40359_2024_2185_MOESM11_ESM.jpg]

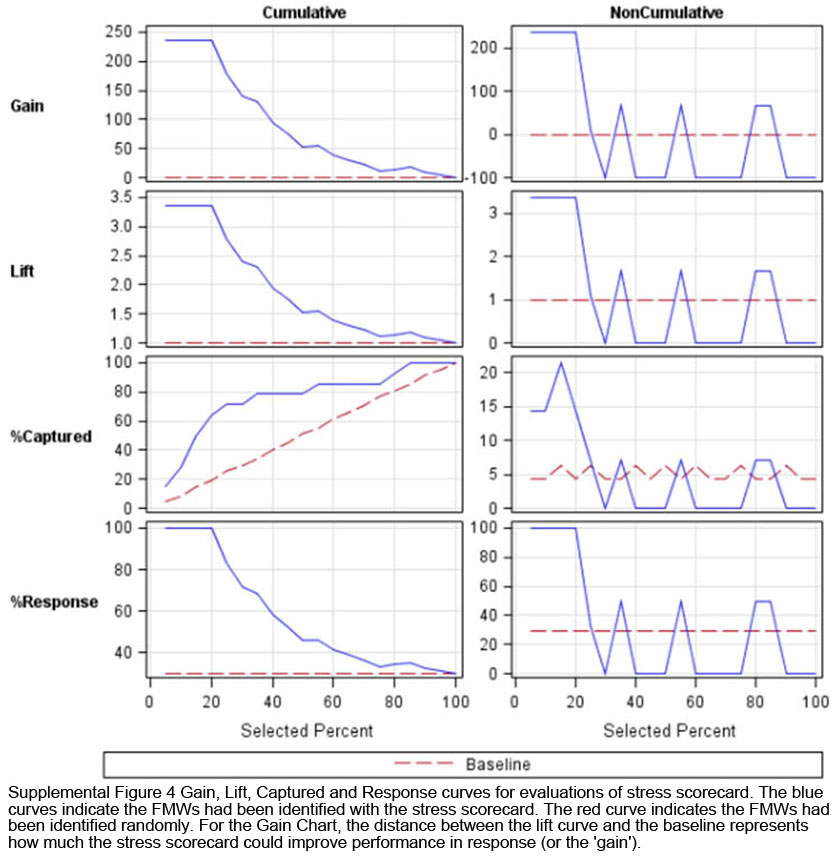

Supplement: Supplementary file 12 — Supplementary Material 12. [file 40359_2024_2185_MOESM12_ESM.jpg]
